# Predicting substrates of the human breast cancer resistance protein using a support vector machine method

**DOI:** 10.1186/1471-2105-14-130

**Published:** 2013-04-15

**Authors:** Eszter Hazai, Istvan Hazai, Isabelle Ragueneau-Majlessi, Sophie P Chung, Zsolt Bikadi, Qingcheng Mao

**Affiliations:** 1 Virtua Drug Ltd., Csalogany Street 4, Budapest H-1015, Hungary; 2 Department of Pharmaceutics, School of Pharmacy, University of Washington, Box 357610, Seattle, Washington 98195, USA

**Keywords:** Breast cancer resistance protein, Support vector machine, SVM, ATP-binding cassette, ABC transporter, *in silico* prediction, Substrate, BCRP, ABCG2

## Abstract

**Background:**

Human breast cancer resistance protein (BCRP) is an ATP-binding cassette (ABC) efflux transporter that confers multidrug resistance in cancers and also plays an important role in the absorption, distribution and elimination of drugs. Prediction as to if drugs or new molecular entities are BCRP substrates should afford a cost-effective means that can help evaluate the pharmacokinetic properties, efficacy, and safety of these drugs or drug candidates. At present, limited studies have been done to develop *in silico* prediction models for BCRP substrates.

In this study, we developed support vector machine (SVM) models to predict wild-type BCRP substrates based on a total of 263 known BCRP substrates and non-substrates collected from literature. The final SVM model was integrated to a free web server.

**Results:**

We showed that the final SVM model had an overall prediction accuracy of ~73% for an independent external validation data set of 40 compounds. The prediction accuracy for wild-type BCRP substrates was ~76%, which is higher than that for non-substrates. The free web server (http://bcrp.althotas.com) allows the users to predict whether a query compound is a wild-type BCRP substrate and calculate its physicochemical properties such as molecular weight, logP value, and polarizability.

**Conclusions:**

We have developed an SVM prediction model for wild-type BCRP substrates based on a relatively large number of known wild-type BCRP substrates and non-substrates. This model may prove valuable for screening substrates and non-substrates of BCRP, a clinically important ABC efflux drug transporter.

## Background

Human breast cancer resistance protein (BCRP, gene symbol *ABCG2*) is an ATP-binding cassette (ABC) efflux drug transporter [1,2]. BCRP is one of the ABC transporters that confer resistance to a large number of structurally and chemically unrelated chemotherapeutic agents through ATP hydrolysis-dependent efflux transport of these drugs [2]. The substrates of BCRP have been rapidly expanding to include not only chemotherapeutics such as mitoxantrone, topotecan and imatinib, but also non-chemotherapeutic drugs such as prazosin, glyburide, nitrofurantoin and statins as well as non-therapeutic compounds such as dietary flavonoids, porphyrins, estrone 3-sulfate, and the dietary carcinogen 2-amino-1-methyl-6-phenylimidazo[4,5-b]pyridine [1,2]. BCRP is also highly expressed in organs important for the absorption (the small intestine), elimination (the liver and kidney), and distribution (the blood–brain and placental barriers) of drugs and xenobiotics [3], and has recently been recognized by the FDA as one of the most important drug transporters involved in clinically relevant drug disposition and drug-drug interactions [4]. Due to the clinical importance of BCRP in drug resistance and drug disposition, it should be of high value to develop cost-effective methods for evaluation of transport of drugs or drug candidates by BCRP so that the pharmacokinetics, efficacy, safety, and tissue levels of these compounds may be predicted. One of such methods would be the development of *in silico* models for prediction of BCRP substrates.

Indeed, in the recent years, *in silico* prediction models have emerged into the pipeline of drug discovery which allow initial screening and selection of promising compounds from chemical libraries and large databases. In addition, these models could provide information concerning the mechanism of protein-ligand interactions. *In silico* methods for prediction of protein-ligand interactions including transport characteristics can be divided into ligand-based and protein structure-based approaches. With protein structure-based methods such as molecular docking, structures and physicochemical characteristics of an intermolecular complex formed between interacting protein and ligand could be predicted if high resolution structures of both the protein and the ligand under question are available. High resolution structures of BCRP have not been resolved. Homology models of BCRP have recently been developed and await further experimental validation [1,5]. Although these homology models can be used for docking calculations and interpretation of biochemical data, results obtained are unlikely reliable for drug design and screening. In contrast, ligand-based methods based on structural similarity of ligands to known substrates generally yield much greater prediction accuracies than protein structure-based methods.

Among ligand-based methods, one common approach is to develop quantitative structure-activity relationship models (SAR and QSAR). The objective of SAR and QSAR analysis is to establish a correlation between descriptors which represent information of molecular structures of ligands and biological activities for a series of biologically and structurally characterized compounds. Various SAR and QSAR models for BCRP inhibitors have been published [6-8]. Several SAR and QSAR studies suggest that lipophilicity of ligands is a good predictor for BCRP inhibition [9-11], but other studies argue that this property is not significant [12,13]. A planar structure of inhibitors seems to be necessary for binding to the active site of BCRP [9,14,15]. With respect to prediction of BCRP substrates, only one SAR study of camptothecin analogues revealed that hydrogen bond formation might be important for substrate recognition by BCRP [16]. One common feature of these SAR and QSAR models is that these models are usually built using a congeneric series of molecules and thus may not be valid for other classes of compounds. For this reason, more sophisticated techniques are required for classification of BCRP ligands.

Another ligand-based approach is to use statistical learning methods to predict features based on properties of examples, and compounds of any chemical structures can be used. Of these methods, the support vector machine (SVM) method is most frequently used and has proved valuable in a wide range of applications. SVM has gained popularity in the chemo- and bioinformatics field due to its ability to classify objects into two classes based on their structural features. In particular, the SVM method was useful for classification of molecules as substrates or non-substrates of enzymes or transporters. For example, several studies have been reported for prediction of substrates and non-substrates of P-glycoprotein (P-gp) using SVM with generally greater than 70% prediction accuracies [17-20]. Zhong et al. recently reported a genetic algorithm-conjugate gradient-support vector machine (GA-CG-SVM) procedure for prediction of BCRP substrates and non-substrates [21]. Although these studies are highly valuable, the scientific community has no open access to most of these published *in silico* models. There are a few SVM-based free web servers for predicting substrates and non-substrates of certain enzymes and transporters. For example, Mishra et al. reported a web server for cytochrome P450 enzymes [22], and our laboratories published a free web server for prediction of P-gp substrates and non-substrates using the SVM method (http://pgp.althotas.com) [20].

Therefore, in the present study, we have compiled a relatively large data set of BCRP substrates and non-substrates collected from literature and developed an SVM-based *in silico* model for prediction of wild-type BCRP substrates and non-substrates. This prediction model has been integrated into a free web server (http://bcrp.althotas.com) which allows the users to predict the capability of wild-type BCRP to transport the query ligands and calculate their physiochemical properties including molecular weight, logP value, and polarizability.

## Methods

### Data set

All known wild-type BCRP substrates and non-substrates used in this study were taken from published data in the literature. Information for some of these compounds in the data set was obtained through searching the University of Washington Metabolism & Transport Drug Interaction Database (http://www.druginteractioninfo.org/). This data set is based on results of *in vitro* transport assays such as the membrane vesicle uptake assay, the efflux assay using intact mammalian cells over-expressing BCRP, and transwell transport assay using MDCKII/BCRP cells. Results from *in vitro* drug resistance assays were also used. However, results from drug-stimulated ATPase assays were not used because many substrates do not stimulate ATPase activity of BCRP. In the case of conflicting evidence, only the results confirmed by at least two independent studies were accepted. This data set contains 164 BCRP substrates and 99 non-substrates with highly diverse chemical structures. We noticed that 60 out of the 164 substrates had multiple reports. However, only about 9 out of the 99 non-substrates had multiple reports. It is worth noting that the drug-selected BCRP mutants with amino acid substitutions at position 482 exhibit altered substrate specificity. For example, doxorubicin, rhodamine 123 and LysoTracker Green are substrates of the mutant R482G or R482T, but cannot be efficiently transported by wild-type BCRP [23-25]. Therefore, such compounds were classified as non-substrates of wild-type BCRP which was the subject of this study. Of the 263 compounds (164 substrates and 99 non-substrates), 223 compounds (139 substrates and 84 non-substrates) were randomly used in the training and test subsets in various training/test ratios, and 40 compounds (25 substrates and 15 non-substrates) were defined as the independent external validation subset. All compounds are listed in Additional file 1: Table S1. The chemical structures of all these molecules are shown in two sdf files provided as Additional files 2 and 3 which can be viewed using the free MarvinView software (http://www.chemaxon.com/products/marvin/marvinview/).

### Support vector machine (SVM)

The SVM method we used in this study is essentially the same as previously described [20]. Briefly, the standard procedure of classification by SVM can be divided into four stages. In the first stage, all compounds in the data set were defined as substrates and non-substrates of wild-type BCRP. Then, the molecules were characterized using molecular descriptors. The data set was then split into the training and test subsets, and an independent external validation subset was also created. In the second stage, the compounds in the training set were presented as points in a high-dimensional space according to their molecular descriptors. In this high-dimensional space, a hyperplane was determined to separate objects into substrate and non-substrate groups. Since various hyperplanes allow separation of objects, a hyperplane that maximizes the margin needs to be constructed. In the third stage, the models constructed using the training data set were used to calculate prediction accuracy for a test set to evaluate the models. Finally, the models were validated using the independent external data set.

Chemical structures of all wild-type BCRP substrates or non-substrates used in this study were downloaded from the PubChem Database (http://pubchem.ncbi.nlm.nih.gov). Some compounds were extracted from the original publications and redrawn by means of MarvinView (ChemAxon, Budapest, Hungary). All molecules were subject to geometry optimization using the Molconvert software (ChemAxon, Budapest, Hungary), which applies the Dreiding molecular mechanics force field, and to calculation of the Gasteiger partial charges [26]. The DragonX software (http://www.talete.mi.it) was used to calculate a total of 3250 molecular descriptors for each molecule. The descriptors with more than 80% zero values and too small standard deviation values (less than 3%) were eliminated. The Libsvm software (http://www.csie.ntu.edu.tw/~cjlin/libsvm/) was then used for SVM calculations. Linear, polynomial, and radial basis function (RBF) kernels were tested in this study. RBF is calculated using the equation K(xi, xj) = exp(−γ||xi-xj||^2^), where γ is a kernel parameter, xi and xj are instance label pairs, and K is the kernel function. The prediction power of SVM is greatly influenced by the selection of kernel, the kernel parameter γ, and soft margin parameter C.

The best combination of C and γ was selected by a grid-search with exponentially growing sequences of C and γ. Each combination of parameter choices was checked, and the parameters with the best validation accuracy were selected. After the best parameters C and γ were found, the whole training set was trained again to generate the final model. The feature selection tool fselect.py (http://www.csie.ntu.edu.tw/~cjlin/libsvmtools) provided by the Libsvm developer was used to measure the relative importance of each feature. For each feature, an F-score can be calculated using fselect.py. Generally, the larger the F-score, the more likely the feature is discriminative. Therefore, F-score was used as a feature selection criterion. Features with high F-scores were selected and then SVM was applied. High F-score features were gradually added until the validation accuracy decreased. Descriptors were checked for their correlation. Among the descriptors with a correlation of 0.9, the descriptors with higher F-scores were kept for further SVM calculations. Prediction power of the above-described SVM method was evaluated based on the number of true positive (TP), true negative (TN), false positive (FP), and false negative (FN) predictions. Additional parameters that are widely used, namely accuracy (ACC), sensitivity (SE), specificity (SP), and the Matthews correlation coefficient (MCC), were also calculated using the following equations:

$$\begin{array}{l} \;\mathrm{ACC}=\left[\left(\mathrm{TP}+\mathrm{TN}\right)/\left(\mathrm{TP}+\mathrm{TN}+\mathrm{FP}+\mathrm{FN}\right)\right]\times 100\\ \;\mathrm{SE}=\left[\mathrm{TP}/\left(\mathrm{TP}+\mathrm{FN}\right)\right]\times 100\\ \;\mathrm{SP}=\left[\mathrm{TN}/\left(\mathrm{FP}+\mathrm{TN}\right)\right]\times 100\\ \mathrm{MCC}=\left(\mathrm{TP}\times \mathrm{TN}-\mathrm{FP}\times \mathrm{FN}\right)/\left[\left(\mathrm{TP}+\mathrm{FN}\right)\right)\left(\mathrm{TP}+\mathrm{FP}\right)\\ \;{\left(\left(\mathrm{TN}+\mathrm{FP}\right)\left(\mathrm{TN}+\mathrm{FN}\right)\right]}^{1/2} \end{array}$$

### The web server

The best prediction model generated using the SVM method described above has been integrated into a free web server (http://bcrp.althotas.com). This web server allows the users to predict as to whether a query compound is likely to be a BCRP substrate. The chemical structure of the query compounds can be uploaded or drawn in by the users using the built-in Chemaxon Marvin Java applet. The web server is linked to PubChem so that any query compounds can be directly retrieved with text search. Any compounds of interest can be searched by their names, uploaded in PDB, mol, mol2, hin, or SMILES format or drawn in using a Marvin applet by the users. Structural conversions and 3-dimensional geometry optimization by the Dreiding method are carried out using the Molconvert software. Two-dimensional and 3-dimensional molecular descriptors are calculated using the DragonX software.

## Results and discussion

Since SVM tends to find a linear separating hyperplane with the maximal margin in a high-dimensional space by using a penalty parameter of the error term and a kernel function, we first investigated the influence of kernel function on the performance parameters. SVM prediction performance parameters of 100 runs with different kernel functions (linear, polynomial, and RBF) are provided in Table 1. The data shown in Table 1 were obtained using a training set of 167 compounds, a test set of 56 compounds, and an external validation set of 40 compounds. The test set was used for choosing the best kernel function, and test performances were used as the criteria for selecting the best kernel. It should be emphasized that the external validation set was not used in the model building steps. It appeared that polynomial kernel function produced generally lower prediction accuracy compared to linear kernel function and RBF. Although performance parameters associated with linear and RBF kernels were comparable, RBF provided slightly better prediction results. This is consistent with a general practice that RBF is the most popular choice of kernel function in SVM. Based on results of this preliminary evaluation, only RBF was used in further calculations.

**Table 1:** The mean values of SVM prediction performance parameters of 100 runs using various kernels

**Kernel**	**Category**	**ACC**	**SE**	**SP**	**MCC**
Linear	Training	80.7	90.4	64.8	0.581
	Test	68.8	82.1	46.6	0.312
	External	70.7	77.6	59.1	0.375
Polynomial	Training	79.2	96.4	50.7	0.506
	Test	65.8	86.8	30.8	0.198
	External	66.1	81.9	39.8	0.174
RBF	Training	84.5	93.8	69.1	0.665
	Test	69.7	83.3	47.2	0.332
	External	70.9	77.7	59.6	0.382

ACC, accuracy (overall prediction accuracy); SP, specificity (prediction accuracy for the non-substrates); SE, sensitivity (prediction accuracy for the substrates); MCC, the Matthews correlation coefficient (a more balanced prediction parameter than ACC). The external data set was only used to validate the prediction power of the models constructed, and was not used for model selection.

Due to the limited number of currently known wild-type BCRP substrates and non-substrates, if more compounds are used in the training set, fewer compounds can be used in the test set, likely resulting in less reliable test prediction outcome. Therefore, we next investigated the influence of the number of compounds in the training and test sets on prediction accuracy. The results of SVM calculations performed with varying training/test set ratios are shown in Table 2. Overall, we did not observe significant differences in the performance parameters with different training/test ratios. However, the MCC values at the training/test ratios of 0.75/0.25; 0.70/0.30 and 0.60/0.40 appeared to be comparable and slightly better than those at other ratios. Similar to the kernel selection, only the test and training data sets were used for model construction, and the external validation data set was only used for validation of the models constructed. Thus, the training/test ratio of 0.75/0.25 was chosen for further calculations in order to maximize the chemical space occupied by the molecules in the training set.

**Table 2:** Performance parameters of 100 runs using various ratios of training/test sets

**Training/Test ratio**	**Category**	**ACC**	**SE**	**SP**	**MCC**
0.5/0.5	Training	83.8	94.6	65.8	0.649
	Test	67.8	82.2	44.0	0.288
	External	69.5	78.3	54.7	0.344
0.6/0.4	Training	85.1	94.7	69.1	0.678
	Test	69.0	83.2	45.5	0.315
	External	70.8	79.0	57.1	0.372
0.7/0.3	Training	83.4	93.3	67.1	0.642
	Test	71.1	84.2	49.1	0.360
	External	70.8	78.4	58.1	0.375
0.75/0.25	Training	84.5	93.8	69.1	0.665
	Test	69.7	83.3	47.2	0.332
	External	70.9	77.7	59.6	0.382
0.8/0.2	Training	83.6	93.5	67.1	0.644
	Test	70.4	83.6	48.7	0.35
	External	70.6	77.5	59.1	0.376
0.85/0.15	Training	82.9	93.5	65.1	0.627
	Test	70.3	85.1	46.4	0.347
	External	70.9	78.5	58.1	0.376

The total number of molecules used in the training and test data sets were 223. The number of molecules in the external validation data set was 40. ACC, accuracy (overall prediction accuracy); SP, specificity (prediction accuracy for the non-substrates); SE, sensitivity (prediction accuracy for the substrates); MCC, the Matthews correlation coefficient (a more balanced prediction parameter than ACC). The external data set was only used to validate the prediction power of the models constructed, and was not used for model selection.

There is no general rule regarding selection of the best SVM prediction model. The run that provides the highest prediction accuracy for the training set may be selected. However, such an approach could be misleading because a model with the highest prediction accuracy (or Matthews correlation coefficient) for a training set does not necessarily produce the highest prediction accuracy (or Matthews correlation coefficient) neither for the test set nor for the independent external validation set due to a phenomenon called overfitting. It is therefore necessary to consider prediction characteristics of both the training and test sets when the best SVM model is to be selected. In our study for prediction of P-gp substrates [20], we proposed the following approach. First, the differences in prediction accuracy between the training and test sets were calculated, and the models with the smallest difference were taken into account. Second, of the models with the smallest difference in prediction accuracy between the training and test sets, those built with the smallest number of molecular descriptors were considered because inclusion of too many descriptors may again produce overfitted models and the inclusion of unnecessary or irrelevant descriptors creates noise in the model. We showed that classification of individual compounds in the independent external validation set as substrates or non-substrates of P-gp was very similar among the potentially best models [20]. Using the same approach, the best SVM prediction model was selected for substrates of wild-type BCRP and the prediction performance parameters of the selected model are shown in Table 3. The selected model showed an overall prediction accuracy of ~73% for the external validation data set. Also, wild-type BCRP substrates were generally predicted with a higher accuracy than non-substrates (Table 3). In the SVM prediction, there is generally a borderline region where the structural properties of substrates and non-substrates are very similar, and therefore compounds in this borderline region cannot be separated by their structural properties alone. In our model, molecules in this borderline region were mainly predicted as substrates. This may contribute to the higher prediction accuracy for substrates.

**Table 3:** Prediction power of the selected SVM model

	**TP**	**FN**	**TN**	**FP**	**ACC**	**SE**	**SP**	**MCC**
Training set	89	15	38	25	76.0	85.6	60.3	0.478
Test set	31	4	11	10	75.0	88.6	52.4	0.448
External set	19	6	10	5	72.5	76.0	66.7	0.422

TP, true positive; TN, true negative; FP, false positive; FN, false negative; ACC, accuracy (overall prediction accuracy); SP, specificity (prediction accuracy for the non-substrates); SE, sensitivity (prediction accuracy for the substrates); MCC, the Matthews correlation coefficient (a more balanced prediction parameter than ACC).

Classification of individual compounds as wild-type BCRP substrates or non-substrates was very similar among the 10 selected models in the first step. Table 4 shows the overlap in classification (i.e. the percentage of compounds that were identically predicted by the compared models) among these 10 models. As can be seen in Table 4, classification of compounds to be wild-type BCRP substrates or non-substrates in different models were highly similar, that is, the overlap values varied between 81.4% and 94.3%, with an average of 88.5%. For example, 85.93% of compounds were predicted to be in the same category (substrates or non-substrates) by the model 87 compared to the model 63, which has the highest prediction accuracy. This finding suggests that, to obtain reliable predictions, selection of the best model is likely not as important as, for example, the compilation of high quality data sets. In this regard, we would like to point out that the data set collected in this study regarding whether a specific compound is a substrate of wild-type BCRP could be obtained using different transport methods. This does not affect the prediction accuracy of our model as long as the different transport methods produce the same result as to if the compound is a BCRP substrate. This is because the SVM model only makes qualitative (substrates or non-substrates), not quantitative (e.g., transport capacity) prediction.

**Table 4:** Overlap of classification in 10 experimental models

**Experimental model**	**98**	**87**	**77**	**63**	**91**	**45**	**42**	**62**	**7**	**73**
98	100	86.31	87.07	86.69	84.41	81.37	87.07	87.07	87.45	86.69
87		100	87.83	85.93	87.45	81.37	87.83	87.07	87.45	90.49
77			100	89.73	94.30	87.45	91.64	92.34	89.73	91.26
63				100	87.83	84.79	88.21	89.73	89.35	88.59
91					100	88.59	88.97	94.30	91.64	92.40
45						100	85.93	88.21	84.79	84.79
42							100	91.64	92.78	92.02
62								100	91.26	91.26
7									100	91.26
73										100

The overall prediction accuracies (ACC) of the 10 experimental models 98, 87, 77, 63, 91, 45, 42, 62, 7, and 73 were 78.33%, 75.29%, 76.81%, 80.23%, 75.67%, 70.34%, 76.05%, 75.29%, 78.71%, and 77.95%, respectively.

Recently, an SVM study based on a different data set was published by Zhong et al. [21] and reported a higher overall prediction accuracy for BCRP substrates and non-substrates (85% for the test set). It should be noted that the compounds used by Zhong et al. were only divided into two sets, namely a training set and a test set, without an independent external validation data set. Also, the test set used by Zhong et al. was not independent when it was used for the selection of the best model. Therefore, their results cannot be directly compared to the data of this study. This is because, besides the training and test sets, we also used an independent external validation data set to evaluate prediction outcome and calculate prediction accuracies of the selected best model. Moreover, certain compounds in the data sets of Zhong et al. were actually the same under different names (e.g., folic acid versus vitamin B9 and daunomycin versus daunorubicin). Additionally, a number of compounds were classified as BCRP substrates (e.g., daunorubicin, rhodamine 123, LysoTracker Green, and epirubicin) by Zhong et al., but as non-substrates in this study as explained in the Methods section.

We found that the final SVM model selected in this study used the molecular descriptors shown in Table 5. That is, the following descriptors were found to be used in the final model: mean information index on atomic composition (AAC), spherosity (SPH), Morse signals, and a mass weighed Gateway descriptor. These descriptors suggest that the 3-dimensional structure of a substrate is likely the determining factor for BCRP/substrate interactions. The results of classification by this SVM model for all compounds used in this study are shown in Additional file 1: Table S1.

**Table 5:** List of molecular descriptors found to be used by the selected SVM model

**Dragon abbreviation**	**Molecular descriptor**
AAC	Mean information index on atomic composition
SPH	spherosity
Mor17m	3D Morse signal 17/weighed by mass
Mor25m	3D Morse signal 25/weighed by mass
R2m	Gateway R autocorrelation of lag2 weighed by mass

In order to make the SVM model publicly available, we developed a free web server (http://bcrp.althotas.com). This web server enables the users to predict if a query compound is a BCRP substrate based on the selected SVM prediction model of this study.

## Conclusions

In summary, BCRP is an ABC drug transporter that confers multidrug resistance in cancers and plays an important role in drug disposition. Therefore, it is important to develop *in silico* prediction models for BCRP substrates that could be used as cost-effective tools for screening of drug candidates in early drug discovery stage and for identification of BCRP substrates among existing drugs so that potential drug-drug interactions may be predicted. In the present study, using a carefully defined and relatively large data set with 263 known wild-type BCRP substrates and non-substrates, we have developed an SVM model for prediction of wild-type BCRP substrates and non-substrates with an overall prediction accuracy of ~73% for an independent external validation data set of 40 compounds. The prediction accuracy for wild-type BCRP substrates was ~76%, which is higher than that for non-substrates. The molecular descriptors used by this SVM model suggest that the 3-dimensional structure of a compound is possibly a predominant factor in determining BCRP/substrate interactions. This SVM prediction model has been integrated into a web server (http://bcrp.althotas.com) which is freely available to the scientific community. We believe that availability of such a prediction model will facilitate drug discovery as well as basic research investigating the role of BCRP in drug transport.

## Abbreviations

ABC: ATP-binding cassette; BCRP: Breast cancer resistance protein; ABCG2: The second member of ABC transporter subfamily G; SVM: Support vector machine; ACC: Accuracy (overall prediction accuracy); SP: Specificity (prediction accuracy for non-substrates); SE: Sensitivity (prediction accuracy for substrates); MCC: Matthews correlation coefficient (a more balanced prediction parameter than ACC); SD: Standard deviation; TP: True positive; FP: False positive; TN: True negative; FN: False negative.

## Competing interests

The authors declare that they have no competing interests.

## Authors’ contributions

EH: data analysis and interpretation, manuscript preparation, and final approval of the version to be published; IH: collection of data sets, data analysis and interpretation, manuscript preparation; SC: collection of data sets and critical revision of the manuscript; IR-M: collection of data sets and critical revision of the manuscript; ZB: data analysis and interpretation, manuscript preparation, and final approval of the version to be published; QM: collection of data sets, data analysis and interpretation, manuscript preparation, and final approval of the version to be published. All authors read and approved the final manuscript.

## Supplementary Material

Additional file 1 All the wild-type BCRP substrates and non-substrates used in this study and classification of these compounds by the selected SVM prediction model developed in this study are shown in the supplemental Table S1. Click here for file

Additional file 2 **The chemical structures of all wild-type BCRP substrates are shown in this sdf file which can be viewed using the free MarvinView software (http://www.chemaxon.com/products/marvin/marvinview/).** The order of molecules in the sdf file is according to the supplementary Table S1. Click here for file

Additional file 3 **The chemical structures of all wild-type BCRP non-substrates are shown in this sdf file which can be viewed using the free MarvinView software (http://www.chemaxon.com/products/marvin/marvinview/).** The order of molecules in the sdf file is according to the supplementary Table S1. Click here for file
