# Supplementary material for: Predicting substrates of the human breast cancer resistance protein using a support vector machine method
Source: BMC Bioinformatics. 2013 Apr 15;14:130. doi: 10.1186/1471-2105-14-130 (PMC3641962; doi:10.1186/1471-2105-14-130)
Supplement: Additional file 1 — All the wild-type BCRP substrates and non-substrates used in this study and classification of these compounds by the selected SVM prediction model developed in this study are shown in the supplemental Table S1. [file 1471-2105-14-130-S1.pdf]

Table S1. List of substrates and non-substrates of wild-type BCRP used in this study

Substrates of wild-type BCRP

Compound	Reference	Predicted property	data set
2-amino-3-methylimidazo[4,5-f] quinoline (IQ)	[1]	S	tr
2-chloro-2'-deoxyadenosine (Cladribine)	[2, 3]	S	tr
3,5-di-O-caffeoylquinic acid	[4]	S	tr
3-amino-1,4-dimethyl-5H-pyrido[4,3-b] indole (Trp-P-1)	[1]	S	tr
4-methylumbelliferone glucuronide	[5]	S	tr
6-MP	[2]	S	tr
7-ethyl-10-hydroxy-camptothecin (SN-38)	[6]	S	tr
7-hydroxy-methotrexate	[7]	S	tr
9-aminocamptothecin	[8]	S	tr
A771726 (an active metabolite of leflunomide)	[9]	S	tr
Acetaminophen glucuronide	[10]	S	tr
Acetaminophen sulfate	[10]	S	tr
Acyclovir	[11]	S	tr
AflatoxinB1	[1]	S	tr
Axitinib	[12]	S	tr
Azidopine	[13]	S	tr
AZT5'-monophosphate	[14, 15]	S	tr
Becatecarin (NSC655649)	[16]	S	tr
Bicalutamide	[17]	S	tr
Biochanin A sulfate	[18]	S	tr
Bisantrene	[19]	S	tr
BN80915 (a difluoro derivative of homocamptothecin)	[20]	S	tr
BODIPY-dihydropyridine	[13]	S	tr
BODIPY-prazosin	[21]	S	tr
BP-3-glucuronide	[22]	S	tr
C-1310	[23]	S	tr
C-1336	[23]	S	tr
C-1584	[23]	S	tr
Canertinib (CI1033)	[24]	S	tr
cGMP	[25]	S	tr
Chlorin e6 (Ce6)	[26]	S	tr
Chlorothiazide	[27]	S	tr
Ciprofloxacin	[28]	S	tr
Clofarabine	[2]	S	tr
Coumestrol	[29]	S	tr
CP-671305	[30]	S	tr
Daidzein sulfate	[31]	S	tr
Dasatinib	[32]	S	tr

E3040-glucuronide	[5]	S	tr
E3040-sulfate	[5]	S	tr
Enrofloxacin	[33]	S	tr
Estrone-3-sulfate (E_1S)	[5, 34]	S	tr
Etoposide	[35]	S	tr
Furosemide	[35]	S	tr
Ganciclovir	[36]	S	tr
Genistein	[29, 37]	S	tr
Genistein sulfate	[31]	S	tr
Gimatecan	[38]	S	tr
Glutathione	[39]	S	tr
Glyburide	[40]	S	tr
GW1843	[41]	S	tr
Harmol sulfate	[10]	S	tr
Hoechst33342	[42]	S	tr
Homocamptothecin	[20]	S	tr
Imatinib mesylate (STI-571)	[43]	S	tr
Iodoarylazidoprazosin (IAAP)	[13]	S	tr
Irinotecan (CPT-11)	[44, 45]	S	tr
J-107088	[46]	S	tr
JNJ-7706621	[47]	S	tr
Kaempferol	[48]	S	tr
Lamivudine (3TC)	[14, 15]	S	tr
Lapatinib	[49]	S	tr
MelQx	[50]	S	tr
Methotrexate-Glu_2	[51, 52]	S	tr
Methotrexate-Glu_3	[51, 52]	S	tr
Mitoxantrone	[19, 21]	S	tr
NB-506	[46]	S	tr
N-desethyl sunitinib	[53]	S	tr
NSC107392	[54]	S	tr
NSC349156	[54]	S	tr
NSC73306	[55]	S	tr
Ofloxacin	[28]	S	tr
Pantoprazole	[56]	S	tr
Pemetrexed	[57]	S	tr
PMEA	[3]	S	tr
Prazosin	[19]	S	tr
Resveratrol-3-sulfate	[58]	S	tr
Riboflavin (VitaminB2)	[59]	S	tr
SN-355	[60]	S	tr
Sorafenib	[61]	S	tr
Sulfasalazine	[62]	S	tr
Sunitinib	[63, 64]	S	tr
Taurocholic acid	[65]	S	tr

Taurolithocholic acid-3-sulfate (TLC-S)	[5]	S	tr
Teniposide	[35]	S	tr
Topotecan	[19]	S	tr
Triamterene	[50]	S	tr
Uric acid (urate)	[66]	S	tr
Vemurafenib (PLX4032)	[67]	S	tr
Bupropion	[68]	NS	tr
CdAMP	[2]	NS	tr
Cholic acid	[65]	NS	tr
Cimetidine	[69]	NS	tr
DHEAS	[5]	NS	tr
Docetaxel	[70]	NS	tr
$E_2$17βG	[5, 71]	NS	tr
Erythromycin	[35]	NS	tr
Fluvastatin	[35]	NS	tr
Moxidectin	[72]	NS	tr
Pheophorbide a	[73]	NS	tr
Phytoporphyrin	[74]	NS	tr
Pravastatin	[75]	NS	tr
Pyropheophorbide-amethylester (MMPa)	[26]	NS	tr
Temocapril	[76]	NS	tr
Abacavir (ABC)	[77, 78]	S	te
Albendazole sulfoxide	[79]	S	te
Atorvastatin	[80]	S	te
Belotecan	[81]	S	te
BODIPY-nilotinib	[82]	S	te
Cediranib	[83]	S	te
Cholylglycylamido fluorescein	[65]	S	te
d-Luciferin	[84]	S	te
Edaravone sulfate	[85]	S	te
Erlotinib	[86]	S	te
Grepafloxacin	[87]	S	te
GV196771	[88]	S	te
Harmol glucuronide	[10]	S	te
Leflunomide	[9]	S	te
ME3277	[89]	S	te
Nitrofurantoin	[90]	S	te
Norfloxacin	[28]	S	te
Olmesartan medoxomil	[91]	S	te
Plumbagin	[92]	S	te
PNU-288034	[93]	S	te
Protoporphyrin IX	[94]	S	te
Quercetin	[48]	S	te
Resveratrol	[7]	S	te
Resveratrol-3-glucuronide	[58]	S	te

Rosiglitazone	[95]	S	te
Rosuvastatin	[35]	S	te
SN-398	[60]	S	te
Tandutinib	[96]	S	te
Tomudex	[41]	S	te
Vandetanib	[97]	S	te
Vitamin K3	[92]	S	te
Butyrate	[98]	NS	te
Cerivastatin	[75]	NS	te
Glycocholic acid	[65]	NS	te
Troglitazone sulfate	[99]	NS	te
2-amnio-1-methyl-6-phenylimidazo(4,5-b)pyridine(PhIP)	[100]	S	e
Apigenin glucuronide	[101]	S	e
Befloxatone	[102]	S	e
Benzo[a]pyrene-3-sulfate (BP-3-sulfate)	[22]	S	e
CP-724714	[103]	S	e
Daidzen glucuronide	[31]	S	e
Dantrolene	[50]	S	e
Diclofenac	[104]	S	e
Dipyridamole	[105]	S	e
Gefitinib	[86]	S	e
Genistein glucuronide	[31, 101]	S	e
Indolocarbazole	[106]	S	e
Oxfendazole (fenbandazole-sulfoxide)	[79]	S	e
Pitavastatin	[107]	S	e
Puromycin	[3]	S	e
Riluzole	[108]	S	e
ST1968	[109]	S	e
Symadex (C-1311)	[23]	S	e
Ulifloxacin	[87]	S	e
17β-estradiol sulfate	[34]	NS	e
5-aminolevulinic acid	[26]	NS	e
NSC265473	[54]	NS	e
Phenethyl isothiocyanate (PEITC)	[110]	NS	e
Sphingosine 1-phosphate	[111]	NS	e
TH-337	[112]	NS	e

Non-substrates of wild-type BCRP

**Compound**	**Reference**	**Predicted property**	**Data set**
(99m) Tc-Tetrofosmin	[113]	NS	tr
Aclarubicin	[114]	NS	tr

Aldosterone	[69]	NS	tr
amino BPU	[115]	NS	tr
Bisoprolol	[95]	NS	tr
C1315	[23]	NS	tr
C1379	[23]	NS	tr
C1554	[23]	NS	tr
Calcein	[19]	NS	tr
Calcein-AM	[19]	NS	tr
Cisplatinum	[116]	NS	tr
Curcumin	[117]	NS	tr
Cyclosporin A	[118]	NS	tr
Dexamethasone	[69]	NS	tr
Digoxin	[69]	NS	tr
Doxorubicin	[114]	NS	tr
Edaravone glucuronide	[85]	NS	tr
Epirubicin	[114]	NS	tr
Estrone	[34]	NS	tr
Indinavir	[119]	NS	tr
Ixabepilone	[70]	NS	tr
Metoprolol	[95]	NS	tr
Nelfinavir	[120]	NS	tr
Nivalenol	[121]	NS	tr
Norbuprenorphine	[122]	NS	tr
Phenobarbital	[123]	NS	tr
Phenytoin	[123]	NS	tr
Salinomycin	[124]	NS	tr
Saquinavir	[120]	NS	tr
Sitosterol	[125]	NS	tr
SN-343	[60]	NS	tr
Valporic acid	[123]	NS	tr
Verapamil	[19]	NS	tr
Vinblastine	[19]	NS	tr
Vitamin B12 (Cyanocobalamin)	[59]	NS	tr
Vitamin B6 (Pyridoxine)	[59]	NS	tr
Vitamin C (Ascorbicacid)	[59]	NS	tr
Vitamin K1 (Phylloquinone)	[59]	NS	tr
5-Fluorouracil	[116]	S	tr
Actinomycin D	[35]	S	tr
Ara-C	[126]	S	tr
C1212	[23]	S	tr
C1375	[23]	S	tr
Cefadroxil	[127]	S	tr
Cidofovir	[128]	S	tr
Fenbendazole	[79]	S	tr
Lamotrigine	[123]	S	tr

Leucovorin	[71]	S	tr
LY294002	[129]	S	tr
Metoprine	[116]	S	tr
Midazolam	[130]	S	tr
m-THPC	[26]	S	tr
Paclitaxel	[19]	S	tr
Prodigiosin	[131]	S	tr
Rhodamine 123	[21]	S	tr
S39625	[132]	S	tr
SN-22	[60]	S	tr
SN-348	[60]	S	tr
SN-351	[60]	S	tr
SN-353	[60]	S	tr
SN-397	[60]	S	tr
SN-443	[60]	S	tr
SN-444	[60]	S	tr
(99m)Tc-Sestamibi	[113]	NS	te
C1213	[23]	NS	te
C1266	[23]	NS	te
Corticosterone	[69]	NS	te
Cortisol	[34]	NS	te
Daunorubicin	[21]	NS	te
Primidone	[123]	NS	te
SN-352	[60]	NS	te
Tacrolimus	[118]	NS	te
Vitamin A (Retinol)	[59]	NS	te
Vitamin B1 (Thiamine)	[59]	NS	te
Albendazole	[79]	S	te
Amonafide L-malate	[133]	S	te
Amprenavir	[120]	S	te
C1176	[23]	S	te
C1503	[23]	S	te
C1558	[23]	S	te
Carbamazepine	[123]	S	te
Clonazepam	[123]	S	te
LysoTrackerGreen	[21]	S	te
mmBPU	[115]	S	te
Adefovir	[128]	NS	e
Buprenorphine	[122]	NS	e
Colchicine	[35]	NS	e
Ethosuximide	[123]	NS	e
OH-bupropion	[68]	NS	e
Propranolol	[95]	NS	e
Ritonavir	[120]	NS	e
Sirolimus	[118]	NS	e

Vincristine	[134]	NS	e
Vitamin E (Tocophenol)	[59]	NS	e
C1415	[23]	S	e
Dimethyl benzoylphenylurea (BPU)	[115]	S	e
Nutlin-3a	[135]	S	e
SN-349	[60]	S	e
YM155	[136]	S	e

Abbreviations: **tr** indicates the compounds in the training set used in the selected model of this study. **te** indicates the compounds in the test set used in the selected model. **e** indicates the compounds in the external validation set used in the selected model. **S** and **NS** denote substrates and non-substrates of wild-type BCRP, respectively. The compounds in the external validation set are highlighted with blue shade.

References

1. van Herwaarden AE, Wagenaar E, Karnekamp B, Merino G, Jonker JW, Schinkel AH: **Breast cancer resistance protein (Bcrp1/Abcg2) reduces systemic exposure of the dietary carcinogens aflatoxin B1, IQ and Trp-P-1 but also mediates their secretion into breast milk**. *Carcinogenesis* 2006, **27**(1):123-130.
2. de Wolf C, Jansen R, Yamaguchi H, de Haas M, van de Wetering K, Wijnholds J, Beijnen J, Borst P: **Contribution of the drug transporter ABCG2 (breast cancer resistance protein) to resistance against anticancer nucleosides**. *Mol Cancer Ther* 2008, **7**(9):3092-3102.
3. Takenaka K, Morgan JA, Scheffer GL, Adachi M, Stewart CF, Sun D, Leggas M, Ejendal KF, Hrycyna CA, Schuetz JD: **Substrate overlap between Mrp4 and Abcg2/Bcrp affects purine analogue drug cytotoxicity and tissue distribution**. *Cancer Res* 2007, **67**(14):6965-6972.
4. Farrell TL, Poquet L, Dew TP, Barber S, Williamson G: **Predicting phenolic acid absorption in Caco-2 cells: a theoretical permeability model and mechanistic study**. *Drug Metab Dispos* 2012, **40**(2):397-406.
5. Suzuki M, Suzuki H, Sugimoto Y, Sugiyama Y: **ABCG2 transports sulfated conjugates of steroids and xenobiotics**. *J Biol Chem* 2003, **278**(25):22644-22649.
6. Nakatomi K, Yoshikawa M, Oka M, Ikegami Y, Hayasaka S, Sano K, Shiozawa K, Kawabata S, Soda H, Ishikawa T *et al*: **Transport of 7-ethyl-10-hydroxycamptothecin (SN-38) by breast cancer resistance protein ABCG2 in human lung cancer cells**. *Biochem Biophys Res Commun* 2001, **288**(4):827-832.
7. Breedveld P, Pluim D, Cipriani G, Dahlhaus F, van Eijndhoven MA, de Wolf CJ, Kuil A, Beijnen JH, Scheffer GL, Jansen G *et al*: **The effect of low pH on breast

cancer resistance protein (ABCG2)-mediated transport of methotrexate, 7-hydroxymethotrexate, methotrexate diglutamate, folic acid, mitoxantrone, topotecan, and resveratrol in in vitro drug transport models. *Mol Pharmacol* 2007, **71**(1):240-249.

8. Rajendra R, Gounder MK, Saleem A, Schellens JH, Ross DD, Bates SE, Sinko P, Rubin EH: **Differential effects of the breast cancer resistance protein on the cellular accumulation and cytotoxicity of 9-aminocamptothecin and 9-nitrocamptothecin**. *Cancer Res* 2003, **63**(12):3228-3233.
9. Kis E, Nagy T, Jani M, Molnar E, Janossy J, Ujhellyi O, Nemet K, Heredi-Szabo K, Krajcsi P: **Leflunomide and its metabolite A771726 are high affinity substrates of BCRP: implications for drug resistance**. *Ann Rheum Dis* 2009, **68**(7):1201-1207.
10. Zamek-Gliszczynski MJ, Nezasa K, Tian X, Kalvass JC, Patel NJ, Raub TJ, Brouwer KL: **The important role of Bcrp (Abcg2) in the biliary excretion of sulfate and glucuronide metabolites of acetaminophen, 4-methylumbelliferone, and harmol in mice**. *Mol Pharmacol* 2006, **70**(6):2127-2133.
11. Gunness P, Aleksa K, Koren G: **Acyclovir is a substrate for the human breast cancer resistance protein (BCRP/ABCG2): implications for renal tubular transport and acyclovir-induced nephrotoxicity**. *Can J Physiol Pharmacol* 2011, **89**(9):675-680.
12. Poller B, Iusuf D, Sparidans RW, Wagenaar E, Beijnen JH, Schinkel AH: **Differential impact of P-glycoprotein (ABCB1) and breast cancer resistance protein (ABCG2) on axitinib brain accumulation and oral plasma pharmacokinetics**. *Drug Metab Dispos* 2011, **39**(5):729-735.
13. Shukla S, Robey RW, Bates SE, Ambudkar SV: **The calcium channel blockers, 1,4-dihydropyridines, are substrates of the multidrug resistance-linked ABC drug transporter, ABCG2**. *Biochemistry* 2006, **45**(29):8940-8951.
14. Wang X, Furukawa T, Nitanda T, Okamoto M, Sugimoto Y, Akiyama S, Baba M: **Breast cancer resistance protein (BCRP/ABCG2) induces cellular resistance to HIV-1 nucleoside reverse transcriptase inhibitors**. *Mol Pharmacol* 2003, **63**(1):65-72.
15. Wang X, Nitanda T, Shi M, Okamoto M, Furukawa T, Sugimoto Y, Akiyama S, Baba M: **Induction of cellular resistance to nucleoside reverse transcriptase inhibitors by the wild-type breast cancer resistance protein**. *Biochem Pharmacol* 2004, **68**(7):1363-1370.
16. Robey RW, Obrzut T, Shukla S, Polgar O, Macalou S, Bahr JC, Di Pietro A, Ambudkar SV, Bates SE: **Becatecarin (rebeccamycin analog, NSC 655649) is a transport substrate and induces expression of the ATP-binding cassette transporter, ABCG2, in lung carcinoma cells**. *Cancer Chemother Pharmacol* 2009, **64**(3):575-583.
17. Colabufo NA, Pagliarulo V, Berardi F, Contino M, Inglese C, Niso M, Ancona P, Albo G, Pagliarulo A, Perrone R: **Bicalutamide failure in prostate cancer treatment: involvement of Multi Drug Resistance proteins**. *Eur J Pharmacol* 2008, **601**(1-3):38-42.

18. An G, Morris ME: **The sulfated conjugate of biochanin A is a substrate of breast cancer resistant protein (ABCG2)**. *Biopharm Drug Dispos* 2011, **32**(8):446-457.
19. Litman T, Brangi M, Hudson E, Fetsch P, Abati A, Ross DD, Miyake K, Resau JH, Bates SE: **The multidrug-resistant phenotype associated with overexpression of the new ABC half-transporter, MXR (ABCG2)**. *J Cell Sci* 2000, **113 (Pt 11)**:2011-2021.
20. Bates SE, Medina-Perez WY, Kohlhagen G, Antony S, Nadjem T, Robey RW, Pommier Y: **ABCG2 mediates differential resistance to SN-38 (7-ethyl-10-hydroxycamptothecin) and homocamptothecins**. *J Pharmacol Exp Ther* 2004, **310**(2):836-842.
21. Robey RW, Honjo Y, Morisaki K, Nadjem TA, Runge S, Risbood M, Poruchynsky MS, Bates SE: **Mutations at amino-acid 482 in the ABCG2 gene affect substrate and antagonist specificity**. *Br J Cancer* 2003, **89**(10):1971-1978.
22. Ebert B, Seidel A, Lampen A: **Identification of BCRP as transporter of benzo[a]pyrene conjugates metabolically formed in Caco-2 cells and its induction by Ah-receptor agonists**. *Carcinogenesis* 2005, **26**(10):1754-1763.
23. Bram EE, Adar Y, Mesika N, Sabisz M, Skladanowski A, Assaraf YG: **Structural determinants of imidazoacridinones facilitating antitumor activity are crucial for substrate recognition by ABCG2**. *Mol Pharmacol* 2009, **75**(5):1149-1159.
24. Erlichman C, Boerner SA, Hallgren CG, Spieker R, Wang XY, James CD, Scheffer GL, Maliepaard M, Ross DD, Bible KC *et al*: **The HER tyrosine kinase inhibitor CI1033 enhances cytotoxicity of 7-ethyl-10-hydroxycamptothecin and topotecan by inhibiting breast cancer resistance protein-mediated drug efflux**. *Cancer Res* 2001, **61**(2):739-748.
25. de Wolf CJ, Yamaguchi H, van der Heijden I, Wielinga PR, Hundscheid SL, Ono N, Scheffer GL, de Haas M, Schuetz JD, Wijnholds J *et al*: **cGMP transport by vesicles from human and mouse erythrocytes**. *FEBS J* 2007, **274**(2):439-450.
26. Robey RW, Steadman K, Polgar O, Bates SE: **ABCG2-mediated transport of photosensitizers: potential impact on photodynamic therapy**. *Cancer Biol Ther* 2005, **4**(2):187-194.
27. Beery E, Rajnai Z, Abonyi T, Makai I, Bansaghi S, Erdo F, Sziraki I, Heredi-Szabo K, Kis E, Jani M *et al*: **ABCG2 modulates chlorothiazide permeability in vitro - characterization of the interaction**. *Drug Metab Pharmacokinet* 2011.
28. Merino G, Alvarez AI, Pulido MM, Molina AJ, Schinkel AH, Prieto JG: **Breast cancer resistance protein (BCRP/ABCG2) transports fluoroquinolone antibiotics and affects their oral availability, pharmacokinetics, and milk secretion**. *Drug Metab Dispos* 2006, **34**(4):690-695.
29. Enokizono J, Kusuhara H, Sugiyama Y: **Effect of breast cancer resistance protein (Bcrp/Abcg2) on the disposition of phytoestrogens**. *Mol Pharmacol* 2007, **72**(4):967-975.
30. Kalgutkar AS, Feng B, Nguyen HT, Frederick KS, Campbell SD, Hatch HL, Bi YA, Kazolias DC, Davidson RE, Mireles RJ *et al*: **Role of transporters in the disposition of the selective phosphodiesterase-4 inhibitor (+)-2-[4-({[2-**

(benzo[1,3]dioxol-5-yloxy)-pyridine-3-carbonyl]-amino}-methyl)-3-fl uoro-phenoxy]-propionic acid in rat and human. *Drug Metab Dispos* 2007, **35**(11):2111-2118.
31. Alvarez AI, Vallejo F, Barrera B, Merino G, Prieto JG, Tomas-Barberan F, Espin JC: **Bioavailability of the glucuronide and sulfate conjugates of genistein and daidzein in breast cancer resistance protein 1 knockout mice**. *Drug Metab Dispos* 2011, **39**(11):2008-2012.
32. Lagas JS, van Waterschoot RA, van Tilburg VA, Hillebrand MJ, Lankheet N, Rosing H, Beijnen JH, Schinkel AH: **Brain accumulation of dasatinib is restricted by P-glycoprotein (ABCB1) and breast cancer resistance protein (ABCG2) and can be enhanced by elacridar treatment**. *Clin Cancer Res* 2009, **15**(7):2344-2351.
33. Pulido MM, Molina AJ, Merino G, Mendoza G, Prieto JG, Alvarez AI: **Interaction of enrofloxacin with breast cancer resistance protein (BCRP/ABCG2): influence of flavonoids and role in milk secretion in sheep**. *J Vet Pharmacol Ther* 2006, **29**(4):279-287.
34. Imai Y, Asada S, Tsukahara S, Ishikawa E, Tsuruo T, Sugimoto Y: **Breast cancer resistance protein exports sulfated estrogens but not free estrogens**. *Mol Pharmacol* 2003, **64**(3):610-618.
35. Lin X, Skolnik S, Chen X, Wang J: **Attenuation of intestinal absorption by major efflux transporters: quantitative tools and strategies using a Caco-2 model**. *Drug Metab Dispos* 2011, **39**(2):265-274.
36. Hu W, Liu W: **Side populations of glioblastoma cells are less sensitive to HSV-TK/GCV suicide gene therapy system than the non-side population**. *In Vitro Cell Dev Biol Anim* 2010, **46**(6):497-501.
37. Imai Y, Tsukahara S, Asada S, Sugimoto Y: **Phytoestrogens/flavonoids reverse breast cancer resistance protein/ABCG2-mediated multidrug resistance**. *Cancer Res* 2004, **64**(12):4346-4352.
38. Marchetti S, Oostendorp RL, Pluim D, van Eijndhoven M, van Tellingen O, Schinkel AH, Versace R, Beijnen JH, Mazzanti R, Schellens JH: **In vitro transport of gimatecan (7-t-butoxyiminomethylcamptothecin) by breast cancer resistance protein, P-glycoprotein, and multidrug resistance protein 2**. *Mol Cancer Ther* 2007, **6**(12 Pt 1):3307-3313.
39. Brechbuhl HM, Gould N, Kachadourian R, Riekhof WR, Voelker DR, Day BJ: **Glutathione transport is a unique function of the ATP-binding cassette protein ABCG2**. *J Biol Chem* 2010, **285**(22):16582-16587.
40. Zhou L, Naraharisetti SB, Wang H, Unadkat JD, Hebert MF, Mao Q: **The breast cancer resistance protein (Bcrp1/Abcg2) limits fetal distribution of glyburide in the pregnant mouse: an Obstetric-Fetal Pharmacology Research Unit Network and University of Washington Specialized Center of Research Study**. *Mol Pharmacol* 2008, **73**(3):949-959.
41. Shafran A, Ifergan I, Bram E, Jansen G, Kathmann I, Peters GJ, Robey RW, Bates SE, Assaraf YG: **ABCG2 harboring the Gly482 mutation confers high-level resistance to various hydrophilic antifolates**. *Cancer Res* 2005, **65**(18):8414-8422.

42. Scharenberg CW, Harkey MA, Torok-Storb B: **The ABCG2 transporter is an efficient Hoechst 33342 efflux pump and is preferentially expressed by immature human hematopoietic progenitors**. *Blood* 2002, **99**(2):507-512.
43. Burger H, van Tol H, Boersma AW, Brok M, Wiemer EA, Stoter G, Nooter K: **Imatinib mesylate (STI571) is a substrate for the breast cancer resistance protein (BCRP)/ABCG2 drug pump**. *Blood* 2004, **104**(9):2940-2942.
44. Rabindran SK, He H, Singh M, Brown E, Collins KI, Annable T, Greenberger LM: **Reversal of a novel multidrug resistance mechanism in human colon carcinoma cells by fumitremorgin C**. *Cancer Res* 1998, **58**(24):5850-5858.
45. Maliepaard M, van Gastelen MA, Tohgo A, Hausheer FH, van Waardenburg RC, de Jong LA, Pluim D, Beijnen JH, Schellens JH: **Circumvention of breast cancer resistance protein (BCRP)-mediated resistance to camptothecins in vitro using non-substrate drugs or the BCRP inhibitor GF120918**. *Clin Cancer Res* 2001, **7**(4):935-941.
46. Komatani H, Kotani H, Hara Y, Nakagawa R, Matsumoto M, Arakawa H, Nishimura S: **Identification of breast cancer resistant protein/mitoxantrone resistance/placenta-specific, ATP-binding cassette transporter as a transporter of NB-506 and J-107088, topoisomerase I inhibitors with an indolocarbazole structure**. *Cancer Res* 2001, **61**(7):2827-2832.
47. Seamon JA, Rugg CA, Emanuel S, Calcagno AM, Ambudkar SV, Middleton SA, Butler J, Borowski V, Greenberger LM: **Role of the ABCG2 drug transporter in the resistance and oral bioavailability of a potent cyclin-dependent kinase/Aurora kinase inhibitor**. *Mol Cancer Ther* 2006, **5**(10):2459-2467.
48. An G, Gallegos J, Morris ME: **The bioflavonoid kaempferol is an Abcg2 substrate and inhibits Abcg2-mediated quercetin efflux**. *Drug Metab Dispos* 2011, **39**(3):426-432.
49. Polli JW, Humphreys JE, Harmon KA, Castellino S, O'Mara MJ, Olson KL, John-Williams LS, Koch KM, Serabjit-Singh CJ: **The role of efflux and uptake transporters in [N-{3-chloro-4-[(3-fluorobenzyl)oxy]phenyl}-6-[5-({[2-(methylsulfonyl)ethyl]amino }methyl)-2-furyl]-4-quinazolinamine (GW572016, lapatinib) disposition and drug interactions**. *Drug Metab Dispos* 2008, **36**(4):695-701.
50. Enokizono J, Kusuhara H, Ose A, Schinkel AH, Sugiyama Y: **Quantitative investigation of the role of breast cancer resistance protein (Bcrp/Abcg2) in limiting brain and testis penetration of xenobiotic compounds**. *Drug Metab Dispos* 2008, **36**(6):995-1002.
51. Chen Z-S, Robey RW, Belinsky MG, Shchaveleva I, Ren X-Q, Sugimoto Y, Ross DD, Bates SE, Kruh GD: **Transport of Methotrexate, Methotrexate Polyglutamates, and 17{beta}-Estradiol 17-({beta}-D-glucuronide) by ABCG2: Effects of Acquired Mutations at R482 on Methotrexate Transport**. *Cancer Res* 2003, **63**(14):4048-4054.
52. Volk EL, Schneider E: **Wild-type breast cancer resistance protein (BCRP/ABCG2) is a methotrexate polyglutamate transporter**. *Cancer Res* 2003, **63**(17):5538-5543.
53. Tang SC, Lankheet NA, Poller B, Wagenaar E, Beijnen JH, Schinkel AH: **P-Glycoprotein (ABCB1) and Breast Cancer Resistance Protein (ABCG2)**

Restrict Brain Accumulation of the Active Sunitinib Metabolite N-Desethyl Sunitinib. *J Pharmacol Exp Ther* 2012, **341**(1):164-173.
54. Deeken JF, Robey RW, Shukla S, Steadman K, Chakraborty AR, Poonkuzhali B, Schuetz EG, Holbeck S, Ambudkar SV, Bates SE: **Identification of compounds that correlate with ABCG2 transporter function in the National Cancer Institute Anticancer Drug Screen**. *Mol Pharmacol* 2009, **76**(5):946-956.
55. Wu CP, Shukla S, Calcagno AM, Hall MD, Gottesman MM, Ambudkar SV: **Evidence for dual mode of action of a thiosemicarbazone, NSC73306: a potent substrate of the multidrug resistance linked ABCG2 transporter**. *Mol Cancer Ther* 2007, **6**(12 Pt 1):3287-3296.
56. Breedveld P, Zelcer N, Pluim D, Sonmezer O, Tibben MM, Beijnen JH, Schinkel AH, van Tellingen O, Borst P, Schellens JH: **Mechanism of the pharmacokinetic interaction between methotrexate and benzimidazoles: potential role for breast cancer resistance protein in clinical drug-drug interactions**. *Cancer Res* 2004, **64**(16):5804-5811.
57. Li L, Sham YY, Bikadi Z, Elmquist WF: **pH-Dependent Transport of Pemetrexed by Breast Cancer Resistance Protein (BCRP)**. *Drug Metab Dispos* 2011.
58. van de Wetering K, Burkon A, Feddema W, Bot A, de Jonge H, Somoza V, Borst P: **Intestinal breast cancer resistance protein (BCRP)/Bcrp1 and multidrug resistance protein 3 (MRP3)/Mrp3 are involved in the pharmacokinetics of resveratrol**. *Mol Pharmacol* 2009, **75**(4):876-885.
59. van Herwaarden AE, Wagenaar E, Merino G, Jonker JW, Rosing H, Beijnen JH, Schinkel AH: **Multidrug transporter ABCG2/breast cancer resistance protein secretes riboflavin (vitamin B2) into milk**. *Mol Cell Biol* 2007, **27**(4):1247-1253.
60. Nakagawa H, Saito H, Ikegami Y, Aida-Hyugaji S, Sawada S, Ishikawa T: **Molecular modeling of new camptothecin analogues to circumvent ABCG2-mediated drug resistance in cancer**. *Cancer Lett* 2006, **234**(1):81-89.
61. Lagas JS, van Waterschoot RA, Sparidans RW, Wagenaar E, Beijnen JH, Schinkel AH: **Breast cancer resistance protein and P-glycoprotein limit sorafenib brain accumulation**. *Mol Cancer Ther* 2010, **9**(2):319-326.
62. Urquhart BL, Ware JA, Tirona RG, Ho RH, Leake BF, Schwarz UI, Zaher H, Palandra J, Gregor JC, Dresser GK *et al*: **Breast cancer resistance protein (ABCG2) and drug disposition: intestinal expression, polymorphisms and sulfasalazine as an in vivo probe**. *Pharmacogenet Genomics* 2008, **18**(5):439-448.
63. Mizuno T, Terada T, Kamba T, Fukudo M, Katsura T, Nakamura E, Ogawa O, Inui K: **ABCG2 421C>A polymorphism and high exposure of sunitinib in a patient with renal cell carcinoma**. *Ann Oncol* 2010, **21**(6):1382-1383.
64. Tang SC, Lagas JS, Lankheet NA, Poller B, Hillebrand MJ, Rosing H, Beijnen JH, Schinkel AH: **Brain accumulation of sunitinib is restricted by P-glycoprotein (ABCB1) and breast cancer resistance protein (ABCG2) and can be enhanced by oral elacridar and sunitinib coadministration**. *Int J Cancer* 2012, **130**(1):223-233.

65. Blazquez AG, Briz O, Romero MR, Rosales R, Monte MJ, Vaquero J, Macias RI, Cassio D, Marin JJ: **Characterization of the role of ABCG2 as a bile acid transporter in liver and placenta**. *Mol Pharmacol* 2012, **81**(2):273-283.
66. Woodward OM, Kottgen A, Coresh J, Boerwinkle E, Guggino WB, Kottgen M: **Identification of a urate transporter, ABCG2, with a common functional polymorphism causing gout**. *Proc Natl Acad Sci U S A* 2009, **106**(25):10338-10342.
67. Mittapalli RK, Vaidhyanathan S, Sane R, Elmquist WF: **Impact of P-glycoprotein (ABCB1) and Breast Cancer Resistance Protein (ABCG2) on the Brain Distribution of a novel B-RAF Inhibitor: Vemurafenib (PLX4032)**. *J Pharmacol Exp Ther* 2012.
68. Hemauer SJ, Patrikeeva SL, Wang X, Abdelrahman DR, Hankins GD, Ahmed MS, Nanovskaya TN: **Role of transporter-mediated efflux in the placental biodisposition of bupropion and its metabolite, OH-bupropion**. *Biochem Pharmacol* 2010, **80**(7):1080-1086.
69. Pavek P, Merino G, Wagenaar E, Bolscher E, Novotna M, Jonker JW, Schinkel AH: **Human breast cancer resistance protein: interactions with steroid drugs, hormones, the dietary carcinogen 2-amino-1-methyl-6-phenylimidazo(4,5-b)pyridine, and transport of cimetidine**. *J Pharmacol Exp Ther* 2005, **312**(1):144-152.
70. Shen H, Lee FY, Gan J: **Ixabepilone, a Novel Microtubule-Targeting Agent for Breast Cancer, Is a Substrate for P-Glycoprotein (P-gp/MDR1/ABCB1) but not Breast Cancer Resistance Protein (BCRP/ABCG2)**. *J Pharmacol Exp Ther* 2011, **337**(2):423-432.
71. Chen ZS, Robey RW, Belinsky MG, Shchaveleva I, Ren XQ, Sugimoto Y, Ross DD, Bates SE, Kruh GD: **Transport of methotrexate, methotrexate polyglutamates, and 17beta-estradiol 17-(beta-D-glucuronide) by ABCG2: effects of acquired mutations at R482 on methotrexate transport**. *Cancer Res* 2003, **63**(14):4048-4054.
72. Perez M, Blazquez AG, Real R, Mendoza G, Prieto JG, Merino G, Alvarez AI: **In vitro and in vivo interaction of moxidectin with BCRP/ABCG2**. *Chem Biol Interact* 2009, **180**(1):106-112.
73. Robey RW, Steadman K, Polgar O, Morisaki K, Blayney M, Mistry P, Bates SE: **Pheophorbide a is a specific probe for ABCG2 function and inhibition**. *Cancer Res* 2004, **64**(4):1242-1246.
74. Robey RW, Fetsch PA, Polgar O, Dean M, Bates SE: **The livestock photosensitizer, phytoporphyrin (phylloerythrin), is a substrate of the ATP-binding cassette transporter ABCG2**. *Res Vet Sci* 2006, **81**(3):345-349.
75. Matsushima S, Maeda K, Kondo C, Hirano M, Sasaki M, Suzuki H, Sugiyama Y: **Identification of the hepatic efflux transporters of organic anions using double-transfected Madin-Darby canine kidney II cells expressing human organic anion-transporting polypeptide 1B1 (OATP1B1)/multidrug resistance-associated protein 2, OATP1B1/multidrug resistance 1, and OATP1B1/breast cancer resistance protein**. *J Pharmacol Exp Ther* 2005, **314**(3):1059-1067.

76. Ohura K, Nozawa T, Murakami K, Imai T: **Evaluation of transport mechanism of prodrugs and parent drugs formed by intracellular metabolism in Caco-2 cells with modified carboxylesterase activity: temocapril as a model case**. *J Pharm Sci* 2011, **100**(9):3985-3994.
77. Pan G, Giri N, Elmquist WF: **Abcg2/Bcrp1 mediates the polarized transport of antiretroviral nucleosides abacavir and zidovudine**. *Drug Metab Dispos* 2007, **35**(7):1165-1173.
78. Giri N, Agarwal S, Shaik N, Pan G, Chen Y, Elmquist WF: **Substrate-dependent breast cancer resistance protein (Bcrp1/Abcg2)-mediated interactions: consideration of multiple binding sites in in vitro assay design**. *Drug Metab Dispos* 2009, **37**(3):560-570.
79. Merino G, Jonker JW, Wagenaar E, Pulido MM, Molina AJ, Alvarez AI, Schinkel AH: **Transport of anthelmintic benzimidazole drugs by breast cancer resistance protein (BCRP/ABCG2)**. *Drug Metab Dispos* 2005, **33**(5):614-618.
80. Keskitalo JE, Zolk O, Fromm MF, Kurkinen KJ, Neuvonen PJ, Niemi M: **ABCG2 polymorphism markedly affects the pharmacokinetics of atorvastatin and rosuvastatin**. *Clin Pharmacol Ther* 2009, **86**(2):197-203.
81. Li H, Jin HE, Kim W, Han YH, Kim DD, Chung SJ, Shim CK: **Involvement of P-glycoprotein, multidrug resistance protein 2 and breast cancer resistance protein in the transport of belotecan and topotecan in Caco-2 and MDCKII cells**. *Pharm Res* 2008, **25**(11):2601-2612.
82. Shukla S, Skoumbourdis AP, Walsh MJ, Hartz AM, Fung KL, Wu CP, Gottesman MM, Bauer B, Thomas CJ, Ambudkar SV: **Synthesis and characterization of a BODIPY conjugate of the BCR-ABL kinase inhibitor Tasigna (nilotinib): evidence for transport of Tasigna and its fluorescent derivative by ABC drug transporters**. *Mol Pharm* 2011, **8**(4):1292-1302.
83. Wang T, Agarwal S, Elmquist WF: **Brain Distribution of Cediranib Is Limited by Active Efflux at the BBB**. *J Pharmacol Exp Ther* 2012.
84. Zhang Y, Bressler JP, Neal J, Lal B, Bhang HE, Laterra J, Pomper MG: **ABCG2/BCRP expression modulates D-Luciferin based bioluminescence imaging**. *Cancer Res* 2007, **67**(19):9389-9397.
85. Mizuno N, Takahashi T, Kusuhara H, Schuetz JD, Niwa T, Sugiyama Y: **Evaluation of the role of breast cancer resistance protein (BCRP/ABCG2) and multidrug resistance-associated protein 4 (MRP4/ABCC4) in the urinary excretion of sulfate and glucuronide metabolites of edaravone (MCI-186; 3-methyl-1-phenyl-2-pyrazolin-5-one)**. *Drug Metab Dispos* 2007, **35**(11):2045-2052.
86. Li J, Cusatis G, Brahmer J, Sparreboom A, Robey RW, Bates SE, Hidalgo M, Baker SD: **Association of variant ABCG2 and the pharmacokinetics of epidermal growth factor receptor tyrosine kinase inhibitors in cancer patients**. *Cancer Biol Ther* 2007, **6**(3):432-438.
87. Ando T, Kusuhara H, Merino G, Alvarez AI, Schinkel AH, Sugiyama Y: **Involvement of breast cancer resistance protein (ABCG2) in the biliary excretion mechanism of fluoroquinolones**. *Drug Metab Dispos* 2007, **35**(10):1873-1879.

88. Polli JW, Baughman TM, Humphreys JE, Jordan KH, Mote AL, Webster LO, Barnaby RJ, Vitulli G, Bertolotti L, Read KD *et al*: **The systemic exposure of an N-methyl-D-aspartate receptor antagonist is limited in mice by the P-glycoprotein and breast cancer resistance protein efflux transporters**. *Drug Metab Dispos* 2004, **32**(7):722-726.
89. Kondo C, Onuki R, Kusuhara H, Suzuki H, Suzuki M, Okudaira N, Kojima M, Ishiwata K, Jonker JW, Sugiyama Y: **Lack of improvement of oral absorption of ME3277 by prodrug formation is ascribed to the intestinal efflux mediated by breast cancer resistant protein (BCRP/ABCG2)**. *Pharm Res* 2005, **22**(4):613-618.
90. Merino G, Jonker JW, Wagenaar E, van Herwaarden AE, Schinkel AH: **The breast cancer resistance protein (BCRP/ABCG2) affects pharmacokinetics, hepatobiliary excretion, and milk secretion of the antibiotic nitrofurantoin**. *Mol Pharmacol* 2005, **67**(5):1758-1764.
91. Yamada A, Maeda K, Kamiyama E, Sugiyama D, Kondo T, Shiroyanagi Y, Nakazawa H, Okano T, Adachi M, Schuetz JD *et al*: **Multiple human isoforms of drug transporters contribute to the hepatic and renal transport of olmesartan, a selective antagonist of the angiotensin II AT1-receptor**. *Drug Metab Dispos* 2007, **35**(12):2166-2176.
92. Shukla S, Wu CP, Nandigama K, Ambudkar SV: **The naphthoquinones, vitamin K3 and its structural analogue plumbagin, are substrates of the multidrug resistance linked ATP binding cassette drug transporter ABCG2**. *Mol Cancer Ther* 2007, **6**(12 Pt 1):3279-3286.
93. Lai Y, Sampson KE, Balogh LM, Brayman TG, Cox SR, Adams WJ, Kumar V, Stevens JC: **Preclinical and clinical evidence for the collaborative transport and renal secretion of an oxazolidinone antibiotic by organic anion transporter 3 (OAT3/SLC22A8) and multidrug and toxin extrusion protein 1 (MATE1/SLC47A1)**. *J Pharmacol Exp Ther* 2010, **334**(3):936-944.
94. Jonker JW, Buitelaar M, Wagenaar E, Van Der Valk MA, Scheffer GL, Scheper RJ, Plosch T, Kuipers F, Elferink RP, Rosing H *et al*: **The breast cancer resistance protein protects against a major chlorophyll-derived dietary phototoxin and protoporphyria**. *Proc Natl Acad Sci U S A* 2002, **99**(24):15649-15654.
95. Solbach TF, Paulus B, Weyand M, Eschenhagen T, Zolk O, Fromm MF: **ATP-binding cassette transporters in human heart failure**. *Naunyn Schmiedebergs Arch Pharmacol* 2008, **377**(3):231-243.
96. Yang JJ, Milton MN, Yu S, Liao M, Liu N, Wu JT, Gan L, Balani SK, Lee FW, Prakash S *et al*: **P-glycoprotein and breast cancer resistance protein affect disposition of tandutinib, a tyrosine kinase inhibitor**. *Drug Metab Lett* 2010, **4**(4):201-212.
97. Azzariti A, Porcelli L, Simone GM, Quatrale AE, Colabufo NA, Berardi F, Perrone R, Zucchetti M, D'Incalci M, Xu JM *et al*: **Tyrosine kinase inhibitors and multidrug resistance proteins: interactions and biological consequences**. *Cancer Chemother Pharmacol* 2010, **65**(2):335-346.

98. Goncalves P, Gregorio I, Martel F: **The short-chain fatty acid butyrate is a substrate of breast cancer resistance protein**. *Am J Physiol Cell Physiol* 2011, **301**(5):C984-994.
99. Enokizono J, Kusuhara H, Sugiyama Y: **Involvement of breast cancer resistance protein (BCRP/ABCG2) in the biliary excretion and intestinal efflux of troglitazone sulfate, the major metabolite of troglitazone with a cholestatic effect**. *Drug Metab Dispos* 2007, **35**(2):209-214.
100. van Herwaarden AE, Jonker JW, Wagenaar E, Brinkhuis RF, Schellens JH, Beijnen JH, Schinkel AH: **The breast cancer resistance protein (Bcrp1/Abcg2) restricts exposure to the dietary carcinogen 2-amino-1-methyl-6-phenylimidazo[4,5-b]pyridine**. *Cancer Res* 2003, **63**(19):6447-6452.
101. Jiang W, Xu B, Wu B, Yu R, Hu M: **UDP-glucuronosyltransferase (UGT) 1A9-overexpressing HeLa cells is an appropriate tool to delineate the kinetic interplay between breast cancer resistance protein (BRCP) and UGT and to rapidly identify the glucuronide substrates of BCRP**. *Drug Metab Dispos* 2012, **40**(2):336-345.
102. Tournier N, Valette H, Peyronneau MA, Saba W, Goutal S, Kuhnast B, Dolle F, Scherrmann JM, Cisternino S, Bottlaender M: **Transport of selected PET radiotracers by human P-glycoprotein (ABCB1) and breast cancer resistance protein (ABCG2): an in vitro screening**. *J Nucl Med* 2011, **52**(3):415-423.
103. Feng B, Xu JJ, Bi YA, Mireles R, Davidson R, Duignan DB, Campbell S, Kostrubsky VE, Dunn MC, Smith AR *et al*: **Role of hepatic transporters in the disposition and hepatotoxicity of a HER2 tyrosine kinase inhibitor CP-724,714**. *Toxicol Sci* 2009, **108**(2):492-500.
104. Lagas JS, van der Kruijssen CM, van de Wetering K, Beijnen JH, Schinkel AH: **Transport of diclofenac by breast cancer resistance protein (ABCG2) and stimulation of multidrug resistance protein 2 (ABCC2)-mediated drug transport by diclofenac and benzbromarone**. *Drug Metab Dispos* 2009, **37**(1):129-136.
105. Zhang Y, Gupta A, Wang H, Zhou L, Vethanayagam RR, Unadkat JD, Mao Q: **BCRP transports dipyridamole and is inhibited by calcium channel blockers**. *Pharm Res* 2005, **22**(12):2023-2034.
106. Nakagawa R, Hara Y, Arakawa H, Nishimura S, Komatani H: **ABCG2 confers resistance to indolocarbazole compounds by ATP-dependent transport**. *Biochem Biophys Res Commun* 2002, **299**(4):669-675.
107. Hirano M, Maeda K, Matsushima S, Nozaki Y, Kusuhara H, Sugiyama Y: **Involvement of BCRP (ABCG2) in the biliary excretion of pitavastatin**. *Mol Pharmacol* 2005, **68**(3):800-807.
108. Milane A, Vautier S, Chacun H, Meininger V, Bensimon G, Farinotti R, Fernandez C: **Interactions between riluzole and ABCG2/BCRP transporter**. *Neurosci Lett* 2009, **452**(1):12-16.
109. De Cesare M, Beretta GL, Tinelli S, Benedetti V, Pratesi G, Penco S, Dallavalle S, Merlini L, Pisano C, Carminati P *et al*: **Preclinical efficacy of ST1976, a novel camptothecin analog of the 7-oxyiminomethyl series**. *Biochem Pharmacol* 2007, **73**(5):656-664.

110. Ji Y, Morris ME: **Membrane transport of dietary phenethyl isothiocyanate by ABCG2 (breast cancer resistance protein)**. *Mol Pharm* 2005, **2**(5):414-419.
111. Takabe K, Kim RH, Allegood JC, Mitra P, Ramachandran S, Nagahashi M, Harikumar KB, Hait NC, Milstien S, Spiegel S: **Estradiol induces export of sphingosine 1-phosphate from breast cancer cells via ABCC1 and ABCG2**. *J Biol Chem* 2010, **285**(14):10477-10486.
112. Meng F, Cai X, Duan J, Matteucci MG, Hart CP: **A novel class of tubulin inhibitors that exhibit potent antiproliferation and in vitro vessel-disrupting activity**. *Cancer Chemother Pharmacol* 2008, **61**(6):953-963.
113. Chen WS, Luker KE, Dahlheimer JL, Pica CM, Luker GD, Piwnica-Worms D: **Effects of MDR1 and MDR3 P-glycoproteins, MRP1, and BCRP/MXR/ABCP on the transport of (99m)Tc-tetrofosmin**. *Biochem Pharmacol* 2000, **60**(3):413-426.
114. Miwa M, Tsukahara S, Ishikawa E, Asada S, Imai Y, Sugimoto Y: **Single amino acid substitutions in the transmembrane domains of breast cancer resistance protein (BCRP) alter cross resistance patterns in transfectants**. *Int J Cancer* 2003, **107**(5):757-763.
115. Rudek MA, Zhao M, Smith NF, Robey RW, He P, Hallur G, Khan S, Hidalgo M, Jimeno A, Colevas AD *et al*: **In vitro and in vivo clinical pharmacology of dimethyl benzoylphenylurea, a novel oral tubulin-interactive agent**. *Clin Cancer Res* 2005, **11**(23):8503-8511.
116. Bram E, Ifergan I, Shafran A, Berman B, Jansen G, Assaraf YG: **Mutant Gly482 and Thr482 ABCG2 mediate high-level resistance to lipophilic antifolates**. *Cancer Chemother Pharmacol* 2006, **58**(6):826-834.
117. Chearwae W, Shukla S, Limtrakul P, Ambudkar SV: **Modulation of the function of the multidrug resistance-linked ATP-binding cassette transporter ABCG2 by the cancer chemopreventive agent curcumin**. *Mol Cancer Ther* 2006, **5**(8):1995-2006.
118. Gupta A, Dai Y, Vethanayagam RR, Hebert MF, Thummel KE, Unadkat JD, Ross DD, Mao Q: **Cyclosporin A, tacrolimus and sirolimus are potent inhibitors of the human breast cancer resistance protein (ABCG2) and reverse resistance to mitoxantrone and topotecan**. *Cancer Chemother Pharmacol* 2006, **58**(3):374-383.
119. Huisman MT, Smit JW, Crommentuyn KM, Zelcer N, Wiltshire HR, Beijnen JH, Schinkel AH: **Multidrug resistance protein 2 (MRP2) transports HIV protease inhibitors, and transport can be enhanced by other drugs**. *AIDS* 2002, **16**(17):2295-2301.
120. Gupta A, Zhang Y, Unadkat JD, Mao Q: **HIV protease inhibitors are inhibitors but not substrates of the human breast cancer resistance protein (BCRP/ABCG2)**. *J Pharmacol Exp Ther* 2004, **310**(1):334-341.
121. Tep J, Videmann B, Mazallon M, Balleydier S, Cavret S, Lecoeur S: **Transepithelial transport of fusariotoxin nivalenol: mediation of secretion by ABC transporters**. *Toxicol Lett* 2007, **170**(3):248-258.
122. Tournier N, Chevillard L, Megarbane B, Pirnay S, Scherrmann JM, Decleves X: **Interaction of drugs of abuse and maintenance treatments with human P-**

glycoprotein (ABCB1) and breast cancer resistance protein (ABCG2). *Int J Neuropsychopharmacol* 2009:1-11.

123. Cerveny L, Pavek P, Malakova J, Staud F, Fendrich Z: **Lack of interactions between breast cancer resistance protein (bcrp/abcg2) and selected antiepileptic agents**. *Epilepsia* 2006, **47**(3):461-468.
124. Lagas JS, Sparidans RW, van Waterschoot RA, Wagenaar E, Beijnen JH, Schinkel AH: **P-glycoprotein limits oral availability, brain penetration, and toxicity of an anionic drug, the antibiotic salinomycin**. *Antimicrob Agents Chemother* 2008, **52**(3):1034-1039.
125. Albrecht C, Elliott JI, Sardini A, Litman T, Stieger B, Meier PJ, Higgins CF: **Functional analysis of candidate ABC transporter proteins for sitosterol transport**. *Biochim Biophys Acta* 2002, **1567**(1-2):133-142.
126. Stam RW, van den Heuvel-Eibrink MM, den Boer ML, Ebus ME, Janka-Schaub GE, Allen JD, Pieters R: **Multidrug resistance genes in infant acute lymphoblastic leukemia: Ara-C is not a substrate for the breast cancer resistance protein**. *Leukemia* 2004, **18**(1):78-83.
127. de Waart DR, van de Wetering K, Kunne C, Duijst S, Paulusma CC, Oude Elferink RP: **Oral availability of cefadroxil depends on ABCC3 and ABCC4**. *Drug Metab Dispos* 2012, **40**(3):515-521.
128. Imaoka T, Kusuhara H, Adachi M, Schuetz JD, Takeuchi K, Sugiyama Y: **Functional involvement of multidrug resistance-associated protein 4 (MRP4/ABCC4) in the renal elimination of the antiviral drugs adefovir and tenofovir**. *Mol Pharmacol* 2007, **71**(2):619-627.
129. Imai Y, Yoshimori M, Fukuda K, Yamagishi H, Ueda Y: **The PI3K/Akt inhibitor LY294002 reverses BCRP-mediated drug resistance without affecting BCRP translocation**. *Oncol Rep* 2012, **27**(6):1703-1709.
130. Franke RM, Lancaster CS, Peer CJ, Gibson AA, Kosloske AM, Orwick SJ, Mathijssen RH, Figg WD, Baker SD, Sparreboom A: **Effect of ABCC2 (MRP2) transport function on erythromycin metabolism**. *Clin Pharmacol Ther* 2011, **89**(5):693-701.
131. Soto-Cerrato V, Llagostera E, Montaner B, Scheffer GL, Perez-Tomas R: **Mitochondria-mediated apoptosis operating irrespective of multidrug resistance in breast cancer cells by the anticancer agent prodigiosin**. *Biochem Pharmacol* 2004, **68**(7):1345-1352.
132. Takagi K, Dexheimer TS, Redon C, Sordet O, Agama K, Lavielle G, Pierre A, Bates SE, Pommier Y: **Novel E-ring camptothecin keto analogues (S38809 and S39625) are stable, potent, and selective topoisomerase I inhibitors without being substrates of drug efflux transporters**. *Mol Cancer Ther* 2007, **6**(12 Pt 1):3229-3238.
133. Burcu M, O'Loughlin KL, Ford LA, Baer MR: **Amonafide L-malate is not a substrate for multidrug resistance proteins in secondary acute myeloid leukemia**. *Leukemia* 2008, **22**(11):2110-2115.
134. Allen JD, Schinkel AH: **Multidrug resistance and pharmacological protection mediated by the breast cancer resistance protein (BCRP/ABCG2)**. *Mol Cancer Ther* 2002, **1**(6):427-434.

135. Zhang F, Throm SL, Murley LL, Miller LA, Steven Zatechka D, Jr., Kiplin Guy R, Kennedy R, Stewart CF: **MDM2 antagonist nutlin-3a reverses mitoxantrone resistance by inhibiting breast cancer resistance protein mediated drug transport**. *Biochem Pharmacol* 2011, **82**(1):24-34.
136. Iwai M, Minematsu T, Li Q, Iwatsubo T, Usui T: **Utility of P-glycoprotein and organic cation transporter 1 double-transfected LLC-PK1 cells for studying the interaction of YM155 monobromide, novel small-molecule survivin suppressant, with P-glycoprotein**. *Drug Metab Dispos* 2011, **39**(12):2314-2320.
